# Comparison of Safety and Efficacy of Avanafil and Tadalafil in the Treatment of Erectile Dysfunction: A Prospective Observational Study

**DOI:** 10.7759/cureus.103529

**Published:** 2026-02-13

**Authors:** Sajal Gupta, Nipun Bansal, Suyog Shetty, Bharat Shejawadkar, Abheesh V Hegde, BM Zeeshan Hameed

**Affiliations:** 1 Department of Urology and Renal Transplantation, Father Muller Medical College, Mangalore, IND; 2 Department of General Surgery, Vardhman Mahavir Medical College and Safdarjung Hospital, Delhi, IND; 3 Department of Urology and Renal Transplantation, Max Super Speciality Hospital, Shalimar Bagh, Delhi, IND; 4 Department of Urology and Renal Transplantation, Kasturba Medical College, Mangalore, IND

**Keywords:** avanafil, efficacy, erectile dysfunction, iief-ef, pde-5 inhibitors, safety, tadalafil

## Abstract

Background

Erectile dysfunction (ED) is considered a condition commonly affecting men, particularly those over 40 years old, with a significant impact on quality of life. Phosphodiesterase-5 (PDE-5) inhibitors, including Avanafil and Tadalafil, are widely used for the treatment of ED. However, there is limited comparative data on their safety and efficacy.

Objective

This study aims to evaluate the safety and efficacy of Avanafil compared to Tadalafil when administered on-demand in patients with ED.

Methods

This prospective observational study was conducted over one year in a tertiary care hospital in India. A total of 106 patients with ED were enrolled and divided into two groups based on the medication prescribed by the treating physician: Group A received Avanafil (100 mg), and Group B received Tadalafil (10 mg). The key primary objective was to measure the change in the International Index of Erectile Function - Erectile Function (IIEF-EF) domain scores at 4, 8, and 12 weeks. Secondary outcomes included changes in other IIEF domains, Sexual Encounter Profile (SEP) scores, and partner satisfaction. Adverse events were recorded to assess safety.

Results

Both Avanafil and Tadalafil significantly improved IIEF-EF scores from baseline. Tadalafil showed superior efficacy, with statistically significant improvements in IIEF-EF scores at 4 weeks (p = 0.008), 8 weeks (p < 0.01), and 12 weeks (p < 0.01) compared to Avanafil. There were no significant differences between the two groups in terms of orgasmic function, sexual desire, intercourse satisfaction, or overall satisfaction. SEP profiles were similar between the groups throughout the study. Adverse effects were mild and comparable between the groups, with no significant differences in the overall incidence of side effects.

Conclusion

Tadalafil demonstrated slightly better efficacy in improving erectile function compared to Avanafil, although both drugs were effective and well-tolerated. The choice of treatment should consider patient preferences and specific clinical contexts, as both medications offer significant benefits with minimal risk of severe adverse effects. Both PDE-5 inhibitors were effective based on patient-reported outcomes, although objective rigidity was not measured.

## Introduction

Erectile dysfunction (ED) is characterized by the failure to achieve or maintain a penile erection that is firm enough for satisfactory sexual intercourse [1]. The prevalence of ED is higher in men over 40 years of age and tends to increase with age and other coexisting health conditions [2]. ED affects approximately 150 million men globally, with an estimated annual incidence of 26 new cases for every 1000 men [3]. The demand for treatments for ED is expected to continue increasing with the anticipated growth in the elderly population. The etiology of ED is frequently complex, involving both physical and psychological factors [1,2].

There are different medical and interventional treatments for addressing ED, such as phosphodiesterase-5 (PDE-5) inhibitors, vacuum erection devices, self-administered penile injection treatments (using vasoactive drugs like alprostadil), and penile prosthesis. Given their ease of use, strong efficacy, and low likelihood of adverse effects, PDE-5 inhibitors have been established as the primary treatment option [4-6].

PDE-5 inhibitors demonstrate high effectiveness, achieving an overall success rate of up to 76% in patients with ED, irrespective of whether or not they have comorbidities. The duration of action of PDE-5 inhibitors varies depending on their respective half-lives, and this factor may affect the choice of medication for the patient [5]. Adverse events may arise in approximately 40% of patients but are generally mild. Headache, nasal congestion, indigestion, and mild changes in vision, such as temporary sensitivity to light or a slight bluish tint to vision, are the most frequently observed side effects [4].

Tadalafil stands out as the most versatile PDE-5 inhibitor available for treating ED. Its extended duration of 17.5 hours is its most distinctive feature, allowing for a wider therapeutic window with on-demand dosing while maintaining consistent plasma levels compared with once-daily dosing. Clinical studies have demonstrated its safety and effectiveness for various causes of ED, including hard-to-treat cases, using both dosing approaches [7].

Avanafil, a second-generation PDE-5 inhibitor, demonstrates a rapid onset of action (starting as quickly as 15 minutes), reaches its peak concentration in the body within 30-45 minutes, and has a duration of action of 3-5 hours. Due to its wide therapeutic index, its on-demand dose may range between 50 and 200 mg depending on effectiveness and/or tolerability [8]. Avanafil has been approved in different countries due to its proven effectiveness and safety in multiple double-blind, placebo-controlled, randomized clinical trials. These trials were conducted in the general population as well as in challenging patient groups, including individuals with diabetes mellitus and those who have had nerve-sparing radical prostatectomy [9-11].

Examining Avanafil and Tadalafil matters clinically due to their distinct pharmacokinetic characteristics. Avanafil is known for its quick onset, ideal for those wanting spontaneity in sexual activities. In contrast, Tadalafil has a longer half-life, allowing for on-demand and daily use, providing versatility. These differences enable doctors to customize treatments based on patient needs and preferences [8].

Despite many years of approval and successful utilization for managing ED, there is only a limited amount of data comparing Avanafil with other PDE-5 inhibitors. We conducted this research to evaluate the safety and efficacy of Avanafil in comparison to Tadalafil when administered on-demand for the treatment of ED.

## Materials and methods

This prospective observational study was conducted over a duration of one year (1/6/2024 to 31/5/2025) at the Department of Urology at a tertiary care hospital (Father Muller Medical College) in India. The study was conducted after receiving approval from the institutional ethics committee (FMIEC/CCM/448/2024) and was formulated according to STROBE (Strengthening the Reporting of Observational Studies in Epidemiology) guidelines [12].

Patients aged 18 years or older with a history of ED for at least three months were included in the study. Additionally, participants needed to have a score of less than 26 on the International Index of Erectile Function - Erectile Function (IIEF-EF) domain. Eligible patients were recruited to the study after obtaining informed consent from the study participants.

Exclusion criteria were comprehensive, excluding individuals who had used PDE-5 inhibitors or any other ED medication within the last three months, had a history of primary hypogonadism, uncontrolled diabetes (HbA1c > 12%), radical prostatectomy, pelvic surgery, or spinal cord injury [13]. Patients with a history of priapism, clinically significant penile deformities or anatomical disorders, cardiac or cerebrovascular disorders, or those with penile implants were also excluded. Furthermore, individuals taking nitric oxide donors, nitrates, alpha-blockers, androgens/anti-androgens, sodium nitroprusside, anticoagulants, antipsychotics/antidepressants, cytochrome P4503A4 inhibitors (such as HIV protease inhibitors, antimycotic agents, erythromycin), herbal products for ED, or amlodipine were not included [13]. Finally, those with a history of alcohol or any drug/substance abuse in the last 12 weeks were also excluded from the study.

The patients were divided into two groups in a 1:1 ratio (non-randomized allocation) depending on the treatment in the form of medication prescribed by the treating physician. Group A received Avanafil 100 mg tablets, to be taken 15 minutes before intercourse, with a maximum dosage of once per day. Group B received a Tadalafil 10 mg tablet, to be taken 30 minutes before intercourse, also with a maximum dosage of once per day. Patients were prescribed Avanafil or Tadalafil as part of routine care and subsequently recruited for observation. Treatment allocation was based on physician preference, and no randomization was performed, which may introduce prescription bias.

A thorough history of each patient was taken during the first visit, including details on any comorbidities, current medications, past surgeries, psychiatric evaluation, and personal and relationship history. A comprehensive examination was also performed at this initial visit. Before initiating any treatment, patients were asked to complete the 15-item validated IIEF questionnaire [14]. Additionally, HbA1c (glycosylated hemoglobin) testing was requested as part of the routine protocol to assess diabetes mellitus status.

Each group received treatment for a maximum period of 12 weeks. The number of drugs consumed and the number of attempts made were recorded for each patient. Follow-up visits were typically scheduled every 4 weeks as part of the routine protocol. The efficacy of the study drugs was evaluated by having patients complete the IIEF questionnaire based on their sexual experiences at the end of 4, 8, and 12 weeks.

After each dose and each attempt at sexual intercourse, patients were asked to record their responses to three specific questions from the Sexual Encounter Profile (SEP) in diary cards: Were you able to achieve at least some erection (some enlargement of the penis) within 15 minutes after drug intake? (modified SEP 1); Were you able to insert your penis into your partner’s vagina? (SEP 2); and did your erection last long enough for you to have successful intercourse? (SEP 3) [13]. For analysis, patients were categorized hierarchically based on the highest level of sexual function achieved: Profile 1 (erection only), Profile 2 (vaginal penetration), and Profile 3 (successful intercourse).

The percentage of successful vaginal penetrations (based on SEP 2), successful intercourse (based on SEP 3), and doses that resulted in some erection within 15 minutes (based on SEP 1) were calculated based on the number of doses taken and sexual attempts made by the patient up to the 4-, 8-, and 12-week visit durations. Additionally, patients were asked to provide feedback from their partner regarding overall satisfaction, which was measured on a scale from 0 to 10 [13].

The primary outcome of the study was the change in the IIEF-EF domain score between the two groups. Secondary efficacy outcomes included the percentage of patients reaching a normal IIEF-EF score, the IIEF score in domains other than EF in the two groups, and the percentages of SEP 1, SEP 2, SEP 3, and partner feedback [14]. All endpoints were evaluated at the end of 4, 8, and 12 weeks of treatment. The safety of the study medications was assessed by recording any adverse events, including headache, myalgia, flushing, nasal congestion, and nausea, that occurred during the course of the study. Any patient reporting chest pain underwent an ECG evaluation and a cardiology consultation, as clinically indicated, to exclude ischemic cardiac events. Partner satisfaction was evaluated using a numeric rating scale specifically for this study. This scale is non-validated and exploratory.

The sample size was calculated using a formula that accounts for a 95% confidence level and 90% power, based on a reference article published by Kumar et al. [13]. The formula used was n = 2 × (Zα + Zβ)² × σ² / (x1 − x2)², where Zα = 1.96 (at 95% confidence interval), Zβ = 1.282 (at 90% power), Σ = 4.5 (pooled standard deviation), (x1-x2)^2^ = 2.0 (mean difference), x1 = 27.1, and x2 = 25.1.

Using this formula, the required sample size was 106 patients, which included participants in both Group A (Avanafil) and Group B (Tadalafil).

Statistical analysis

The data were gathered and recorded in a data collection sheet format using MS Excel (Microsoft Corporation, Redmond, Washington). Mean ± SD or median (IQR) were used to present continuous variables depending on data distribution, which was assessed using the Shapiro-Wilk test and visual inspection of histograms. Frequencies and percentages with a 95% confidence interval were used to express categorical variables. Statistical analysis was conducted using IBM SPSS Statistics for Windows, Version 20 (Released 2011; IBM Corp., Armonk, New York).

A multivariable linear regression model adjusting for baseline IIEF-EF score, age, smoking status, diabetes, and BMI was performed to confirm the robustness of results; however, detailed model outputs are not presented. Continuous and categorical data were compared using Student’s t-tests and chi-square tests, respectively. No formal adjustment for multiple comparisons was performed due to the exploratory nature of secondary outcomes. Missing data were minimal (<5%) and handled using complete-case analysis. Changes in IIEF and SEP outcomes over time were evaluated using repeated-measures ANOVA to account for within-subject correlations across the four assessment points (baseline, 4, 8, and 12 weeks). We examined the main effects of time, group effect, and group × time interaction. Significant overall effects were followed by Bonferroni-adjusted pairwise comparisons between time points. Statistical significance was defined as a two-tailed p < 0.05.

## Results

A total of 106 eligible patients participated in this study, with 53 patients in the Avanafil group and 53 in the Tadalafil group. The mean age of patients in the Avanafil group was 50.5 ± 11.4 years (n = 53), compared to 51.9 ± 10.5 years (n = 53) in the Tadalafil group, with no statistically significant difference between the two groups (P = 0.49). The duration of symptoms was also comparable, with 5.0 ± 3.1 months (n = 53) in the Avanafil group and 5.3 ± 3.1 months (n = 53) in the Tadalafil group (P = 0.57). Moreover, the severity of ED at baseline showed no significant difference between the groups (P = 0.803).

In the Avanafil group, 13 (24.5%, n = 13/53) patients were classified as having mild ED (IIEF-EF score 22-25), 21 (39.6%, n = 21/53) as mild to moderate, 15 (28.3%, n = 15/53) as moderate, and 4 (7.5%, n = 4/53) as severe. In comparison, the Tadalafil group had 10 (18.9%, n = 10/53) patients with mild ED, 25 (47.2%, n = 25/53) with mild to moderate ED, 13 (24.5%, n = 13/53) with moderate ED, and 5 (9.4%, n = 5/53) with severe ED.

These findings suggest that the two groups were well matched in terms of demographic and clinical characteristics at baseline. Baseline characteristics of patients in both groups are described in Table 1.

**Table 1:** Baseline characteristics of the study population Baseline characteristics of the study population. Continuous variables (e.g., age, duration of symptoms, IIEF-EF score) are presented as mean ± SD and were compared using independent samples t-tests. Categorical variables (e.g., history of smoking, comorbidities, ED severity categories) are presented as n (%) and were compared using the chi-square test or Fisher’s exact test where appropriate. A p-value < 0.05 was considered statistically significant. ED: Erectile dysfunction; IIEF-EF: International Index of Erectile Function - Erectile Function; BMI: Body mass index

Variables	Avanafil (n = 53)	Tadalafil (n = 53)	p-value
Age (years)	50.5 ± 11.4	51.9 ± 10.5	0.49
BMI (kg/m²)	25.6 ± 3.0	25.7 ± 2.6	0.78
Duration of symptoms (months)	5.0 ± 3.1	5.3 ± 3.1	0.57
History of smoking	18 (34%)	11 (20.8%)	0.13
Diabetes	14 (26.4%)	14 (26.4%)	0.85
Hypertension	23 (43.4%)	21 (39.6%)	0.9
Erectile function (IIEF-EF) score	17.7 ± 4.3	17.6 ± 4.6	0.914
ED severity at baseline			
Mild (22–25)	13 (24.5%)	10 (18.9%)	0.803
Mild to moderate (17–21)	21 (39.6%)	25 (47.2%)	
Moderate (11–16)	15 (28.3%)	13 (24.5%)	
Severe (1–10)	4 (7.5%)	5 (9.4%)	

As elaborated in Table 2, patients were observed and reassessed at follow-ups every 4 weeks. Both groups took a similar number of pills during each 4-week period, with a median of 5 (range 2-10; n = 53) in the Avanafil group and 5 (range 3-11; n = 53) in the Tadalafil group (p = 0.37). The number of attempts at sexual intercourse was also similar, with a median of 5 (range 3-11; n = 53) attempts in the Avanafil group and 6 (range 2-13; n = 53) attempts in the Tadalafil group (p = 0.34).

**Table 2:** Follow-up characteristics Follow-up characteristics. Median (range) values for pills taken and attempts at intercourse per 4-week period are shown. Between-group comparisons for these non-normally distributed variables were performed using the Mann–Whitney U test. P-values < 0.05 were considered statistically significant.

Variable	Avanafil Group	Tadalafil Group	p-value
Follow-ups (every 4 weeks)			
Pills taken per 4-week period (median, range)	5 (2–10)	5 (3–11)	0.37
Attempts at intercourse per 4-week period (median, range)	5 (3–11)	6 (2–13)	0.34

**Figure 1:**
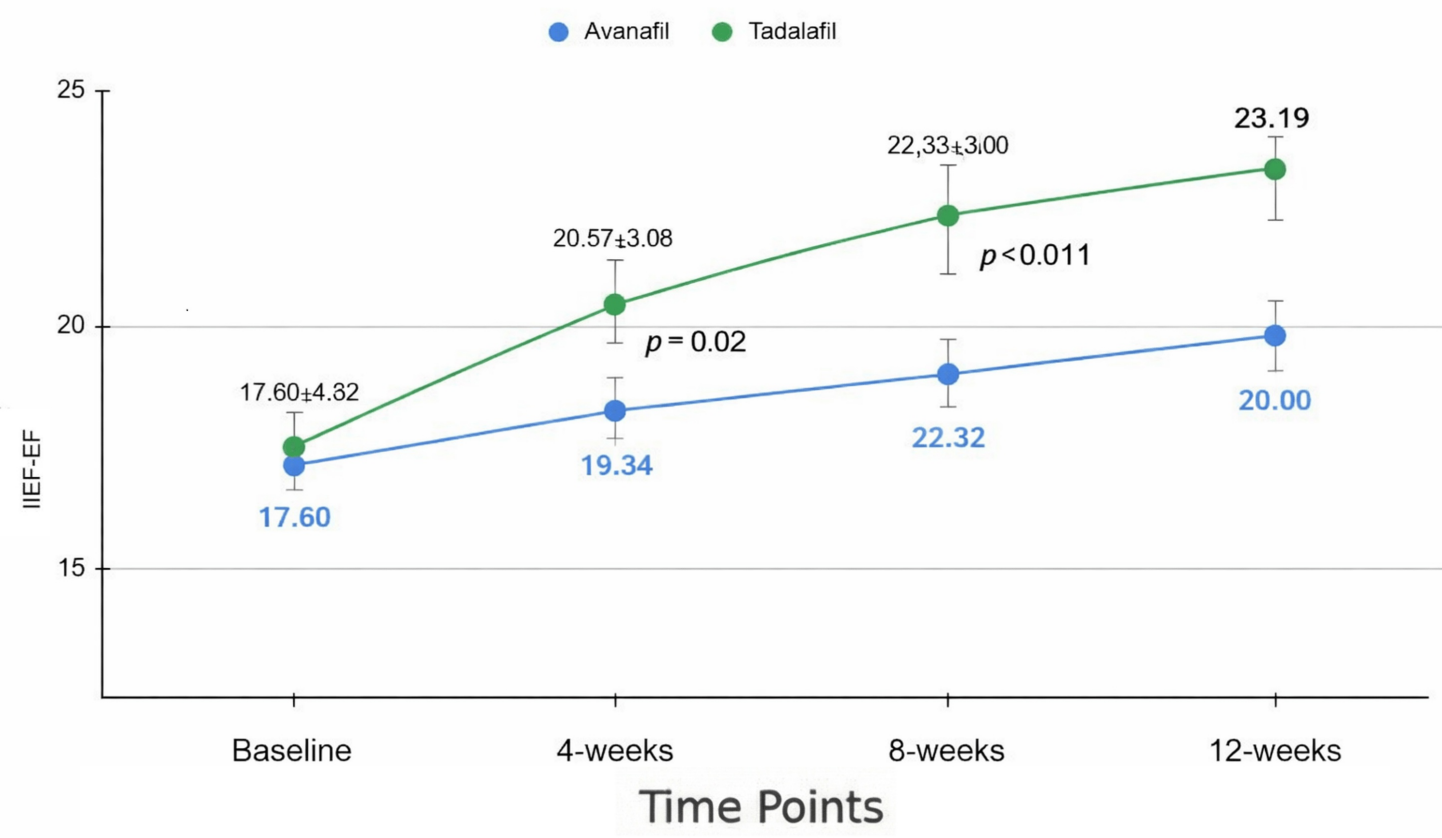
Changes in IIEF-EF scores over time (mean ± SD). Statistical comparisons are shown between time points (p-values) IIEF-EF: International Index of Erectile Function - Erectile Function

In this study, we compared the effects of Avanafil and Tadalafil on various aspects of sexual function over a 12-week period. As depicted in Figure 1, the primary outcome IIEF-EF scores showed comparable baseline values between the Avanafil group (mean ± SD: 17.70 ± 4.32, n = 53) and the Tadalafil group (mean ± SD: 17.60 ± 4.65, n = 53).

By week 4, the Tadalafil group exhibited a statistically significant improvement in IIEF-EF (20.57 ± 4.08, n = 53) compared to the Avanafil group (18.83 ± 3.63, n = 53; p = 0.008). This trend continued through week 8 (22.32 ± 3.50, n = 53 vs. 19.34 ± 3.06, n = 53; p < 0.001) and week 12 (23.19 ± 2.98, n = 53 vs. 20.00 ± 2.87, n = 53; p < 0.001), indicating a superior efficacy of Tadalafil in improving erectile function over time. Using repeated-measures ANOVA, we observed significant improvements in IIEF-EF scores over time in both the Avanafil and Tadalafil groups (overall p < 0.001). Pairwise comparisons adjusted by Bonferroni correction showed significant increases from baseline to 4, 8, and 12 weeks in both groups.

In the multivariable regression model adjusted for age, baseline erectile function (IIEF-EF score), and comorbidities, including the presence of diabetes mellitus, the treatment group was independently associated with a change in IIEF-EF scores. Specifically, assignment to the Tadalafil group was associated with a mean increase in IIEF-EF of β = 2.35 (95% CI: 1.10 to 3.60, p = 0.001). The model's R² was 0.42, indicating that 42% of the variability in the change in IIEF-EF scores was explained by the model. Other covariates (e.g., age, diabetes, and hypertension) did not show statistically significant independent associations with changes in IIEF-EF in this model.

Secondary outcomes included measures of orgasmic function, sexual desire, intercourse satisfaction, overall satisfaction, and partner’s feedback on overall satisfaction. For orgasmic function, there were no significant differences between the groups at any time point, with week 12 scores of 8.36 ± 1.06 (n = 53) for Avanafil and 8.21 ± 1.06 (n = 53; p = 0.47) for Tadalafil. Sexual desire scores were also similar throughout the study, with no significant differences at week 12 (7.89 ± 1.20, n = 53 vs. 7.79 ± 1.25, n = 53; p = 0.69). Intercourse satisfaction, overall satisfaction, and partner’s feedback improved slightly in both groups, with no statistically significant differences observed between Avanafil and Tadalafil at the 12-week follow-up (as shown in Table 3).

**Table 3:** Comparison of IIEF domain scores between Avanafil and Tadalafil over time Comparison of IIEF domain scores between Avanafil and Tadalafil over time. Results are presented as mean ± SD. Between-group comparisons at each time point were performed using independent samples t-tests. Within-group changes over time were evaluated using repeated-measures analysis (e.g., repeated-measures ANOVA or an equivalent nonparametric test if assumptions were not met). P-values < 0.05 indicate statistical significance. IIEF: International Index of Erectile Function; ANOVA: Analysis of variance

Outcome/Time Point	Avanafil (n = 53)	Tadalafil (n = 53)	p-value
	Mean ± SD	Mean ± SD	
Erectile function (IIEF-EF)			
Baseline	17.70 ± 4.32	17.60 ± 4.65	0.91
4 weeks	18.83 ± 3.63	20.57 ± 4.08	0.02
8 weeks	19.34 ± 3.06	22.32 ± 3.50	< 0.01
12 weeks	20.00 ± 2.87	23.19 ± 2.98	< 0.01
p-value (change over time)	0.008	< 0.001	-
Orgasmic function			
Baseline	8.00 ± 1.16	7.70 ± 1.17	0.19
4 weeks	8.25 ± 1.05	7.83 ± 1.12	0.06
8 weeks	8.30 ± 1.07	7.92 ± 1.05	0.07
12 weeks	8.36 ± 1.06	8.21 ± 1.06	0.47
p-value (change over time)	0.098	0.026	-
Sexual desire			
Baseline	7.47 ± 1.40	7.42 ± 1.50	0.84
4 weeks	7.64 ± 1.37	7.47 ± 1.50	0.55
8 weeks	7.77 ± 1.23	7.60 ± 1.39	0.51
12 weeks	7.89 ± 1.20	7.79 ± 1.25	0.69
p-value (change over time)	0.1	0.17	-
Intercourse satisfaction			
Baseline	8.94 ± 2.24	9.08 ± 2.13	0.76
4 weeks	9.89 ± 2.07	10.02 ± 2.09	0.75
8 weeks	10.15 ± 1.90	10.08 ± 2.03	0.84
12 weeks	10.38 ± 1.90	10.26 ± 1.92	0.76
p-value (change over time)	< 0.01	0.003	-
Overall satisfaction			
Baseline	6.45 ± 1.03	6.32 ± 1.12	0.53
4 weeks	6.79 ± 0.93	6.66 ± 1.18	0.52
8 weeks	6.98 ± 0.93	6.89 ± 1.01	0.62
12 weeks	7.15 ± 0.93	7.17 ± 1.07	0.92
p-value (change over time)	0.008	< 0.01	-
Partner’s feedback—overall satisfaction (0–10)			
Baseline	5.17 ± 1.41	5.19 ± 1.37	0.95
4 weeks	5.55 ± 1.23	5.68 ± 1.40	0.61
8 weeks	5.74 ± 1.20	6.06 ± 1.41	0.21
12 weeks	6.17 ± 1.03	6.38 ± 1.23	0.35
p-value (change over time)	< 0.01	< 0.01	-

Both Avanafil and Tadalafil significantly improved erectile function, intercourse satisfaction, overall satisfaction, and partner satisfaction over 12 weeks (p < 0.05 for within-group changes). However, improvement in orgasmic function was observed only among patients on Tadalafil (p = 0.026), and there was no meaningful improvement in sexual desire in either group.

**Figure 2:**
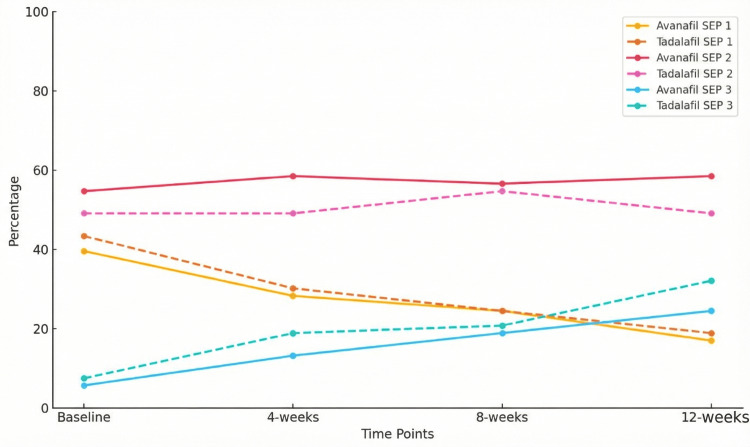
Comparison of SEP profiles over time for Avanafil and Tadalafil SEP: Sexual encounter profile

While comparing the SEP profiles between Avanafil and Tadalafil over a 12-week period, the distributions at baseline were similar, with 21 (39.6%, n = 21/53) patients in the Avanafil group and 23 (43.4%, n = 23/53) in the Tadalafil group categorized in SEP Profile 1, 29 (54.7%, n = 29/53) and 26 (49.1%, n = 26/53) in SEP Profile 2, and 3 (5.7%, n = 3/53) and 4 (7.5%, n = 4/53) in SEP Profile 3, respectively (p = 0.82). By week 4, the proportions in SEP Profile 1 decreased to 15 (28.3%, n = 15/53) for Avanafil and 17 (32.1%, n = 17/53) for Tadalafil, while SEP Profile 3 increased to 7 (13.2%, n = 7/53) and 10 (18.9%, n = 10/53) for Avanafil and Tadalafil, respectively (p = 0.58). At week 8, the distribution remained consistent, with 13 (24.5%, n = 13/53) of patients in SEP Profile 1 for both groups, while SEP Profile 3 increased further to 10 (18.9%, n = 10/53) for Avanafil and 11 (20.8%, n = 11/53) for Tadalafil (p = 0.97). By 12 weeks, SEP Profile 1 decreased further to 9 (17.0%, n = 9/53) for Avanafil and 10 (18.9%, n = 10/53) for Tadalafil, with SEP Profile 3 increasing to 13 (24.5%, n = 13/53) and 17 (32.1%, n = 17/53), respectively (p = 0.60). Throughout the study, SEP Profile 2 remained the predominant category for both treatments, and there were no statistically significant differences between the two groups at any time point, as shown in Table 4 and Figure 2. Partner satisfaction was assessed using a non-validated numeric rating scale.

**Table 4:** Distribution of patients classified into SEP Profiles 1, 2, and 3, over time for Avanafil and Tadalafil Distribution of patients classified into SEP Profiles 1, 2, and 3 over time for Avanafil and Tadalafil. Values are presented as n (%). Between-group comparisons at each time point were performed using the chi-square test. When cell counts were low (expected frequency <5), Fisher’s exact test was used. P-values < 0.05 were considered statistically significant.

Timepoint	Avanafil (n = 53)			Tadalafil (n = 53)			p-value
	SEP Profile 1	SEP Profile 2	SEP Profile 3	SEP Profile 1	SEP Profile 2	SEP Profile 3	
Baseline	21 (39.6%)	29 (54.7%)	3 (5.7%)	23 (43.4%)	26 (49.1%)	4 (7.5%)	0.82
4 weeks	15 (28.3%)	31 (58.5%)	7 (13.2%)	17 (32.1%)	26 (49.1%)	10 (18.9%)	0.58
8 weeks	13 (24.5%)	30 (56.6%)	10 (18.9%)	13 (24.5%)	29 (54.7%)	11 (20.8%)	0.97
12 weeks	9 (17.0%)	31 (58.5%)	13 (24.5%)	10 (18.9%)	26 (49.1%)	17 (32.1%)	0.6

Table 5 shows the incidence of adverse effects over 4, 8, and 12 weeks in patients treated with Avanafil and Tadalafil. At 4 weeks, chest pain was reported by 1 (1.9%, n = 1/53) Avanafil user and 3 (5.7%, n = 3/53) Tadalafil users; at 8 weeks, chest pain was reported by 3 (5.7%, n = 3/53) Avanafil users and 0 (0.0%, n = 0/53) Tadalafil users. By 12 weeks, chest pain was absent in Avanafil users (0.0%, n = 0/53) but reported by 2 (3.8%, n = 2/53) Tadalafil users. All patients reporting chest pain underwent ECG evaluation, which was normal, and symptoms resolved without intervention; none of these patients required discontinuation of the study medication.

**Table 5:** Comparison of the incidence of adverse effects between the two groups Comparison of the incidence of adverse effects between the two groups at 4, 8, and 12 weeks. Values are presented as n (%). Between-group comparisons at each time point were performed using the chi-square test or Fisher’s exact test where appropriate. P-values < 0.05 indicate statistical significance.

Adverse effects	Avanafil 4-weeks	Tadalafil 4-weeks	Avanafil 8-weeks	Tadalafil 8-weeks	Avanafil 12-weeks	Tadalafil 12-weeks
Chest pain	1 (1.9%)	3 (5.7%)	3 (5.7%)	0 (0.0%)	0 (0.0%)	2 (3.8%)
Flushing	4 (7.5%)	6 (11.3%)	5 (9.4%)	2 (3.8%)	3 (5.7%)	3 (5.7%)
Headache	6 (11.3%)	6 (11.3%)	4 (7.5%)	6 (11.3%)	5 (9.4%)	5 (9.4%)
Myalgia	4 (7.5%)	10 (18.9%)	6 (11.3%)	6 (11.3%)	4 (7.5%)	8 (15.1%)
Nasal congestion	6 (11.3%)	1 (1.9%)	2 (3.8%)	5 (9.4%)	5 (9.4%)	1 (1.9%)
Nausea	4 (7.5%)	3 (5.7%)	4 (7.5%)	2 (3.8%)	3 (5.7%)	3 (5.7%)
Total	25 (47.2%)	29 (54.7%)	24 (45.3%)	21 (39.6%)	20 (37.7%)	22 (41.6%)
p-value	0.239		0.341		0.416	

Flushing was more common in Tadalafil users at 4 weeks with 6 (11.3%, n = 6/53) versus 4 (7.5%, n = 4/53) for Avanafil, but decreased over time, with both groups reporting 3 (5.7%, n = 3/53) at 12 weeks. Headache followed a similar pattern in both groups across the study period, and myalgia showed a slightly higher incidence in the Tadalafil group at 12 weeks (8 (15.1%, n = 8/53) vs. 4 (7.5%, n = 4/53) for Avanafil). Nasal congestion was reported more frequently in Avanafil users at 4 weeks (5 (9.4%, n = 5/53) vs. 3 (5.7%, n = 3/53)) and at 12 weeks (4 (7.5%, n = 4/53) vs. 2 (3.8%, n = 2/53)). Nausea remained consistent between the groups at all time points, with similar sample counts and percentages.

**Figure 3:**
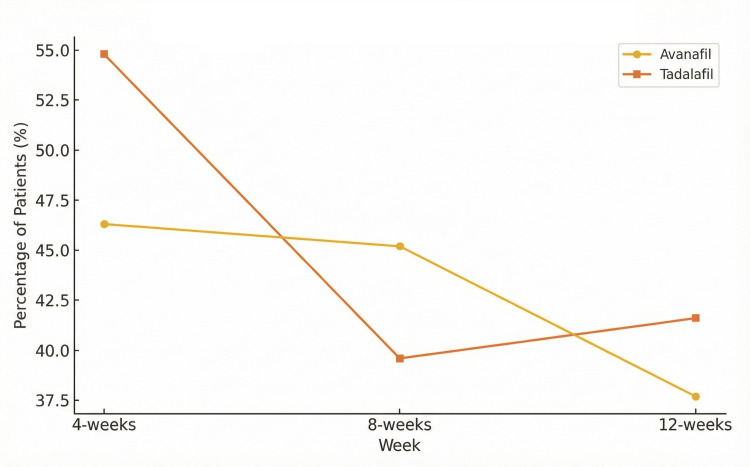
Adverse effects comparison over 12 weeks

Overall, there were no statistically significant differences between the two drugs in the total adverse effects reported, with p = 0.239 (n = 53/53 vs. 53/53 at 4 weeks), p = 0.341 (n = 53/53 vs. 53/53 at 8 weeks), and p = 0.416 (n = 53/53 vs. 53/53 at 12 weeks) (Figure 3).

## Discussion

This research evaluated the safety and effectiveness of Avanafil and Tadalafil for treating ED over 12 weeks. Results show both PDE-5 inhibitors significantly enhanced erectile function, but Tadalafil was associated with greater improvement in IIEF-EF scores in this cohort. Avanafil's quicker onset benefits patients needing immediate readiness, while Tadalafil's extended duration enables spontaneous interactions throughout the day. These differences highlight the need for personalized treatment strategies, as supported by existing pharmacokinetic and patient-preference data [7]. Future studies should include randomized trials with extended follow-up to evaluate long-term effectiveness and adherence. Direct comparisons of all PDE-5 inhibitors under uniform conditions would provide more robust clinical guidance. Our findings confirm the practical applicability of both medications for patient groups with diverse expectations regarding the timing and duration of effects [7]. Although the observed differences in erectile function between the Tadalafil and Avanafil groups were statistically significant, these findings should be interpreted with caution, given the study's observational design and reliance on patient-reported outcomes rather than objective measures.

In terms of secondary outcomes, such as orgasmic function, sexual desire, intercourse satisfaction, and overall satisfaction, both drugs performed similarly, with only modest differences observed. Notably, improvement in orgasmic function was significant only in the Tadalafil group, which may be attributable to its pharmacodynamic properties. The similarity in sexual desire and overall satisfaction across both groups suggests that both medications are effective in enhancing these aspects of sexual function, although neither showed significant superiority.

The proportion of patients classified to have an SEP 1 profile of sexual encounter reduced meaningfully with a concurrent rise in patients with SEP 3. This was observed similarly in both the study groups. This indicates that both Avanafil and Tadalafil are reliable in facilitating sexual activity. However, no statistically significant differences were found between the two groups, suggesting comparable effectiveness in real-world scenarios.

Adverse effects were generally mild and consistent with previous reports on PDE-5 inhibitors. Both drugs had similar safety profiles, with the most common side effects being headache, myalgia, and flushing. Interestingly, while Tadalafil had higher rates of myalgia (15.1% vs 7.5% at 12 weeks), this difference was not statistically significant (p=0.416 for total events). Avanafil showed more frequent cases of nasal congestion. Despite these differences, the overall incidence of adverse effects did not significantly differ between the two groups, affirming the safety of both medications for most patients. Two participants reported transient chest discomfort. Both underwent ECG evaluation with normal results, and symptoms resolved without intervention. Patients were advised to discontinue activity and seek evaluation if symptoms worsened. Importantly, no ischemic cardiac events were identified among patients reporting chest pain, and none required discontinuation of study medication.

To our knowledge, there are no adequately powered prospective randomized trials directly comparing Avanafil and Tadalafil. Available evidence largely consists of individual trials in which each drug has been compared against sildenafil rather than against the others. A randomized controlled trial demonstrated the efficacy and safety of Avanafil compared with sildenafil [13], while a systematic review and meta-analysis reported comparable efficacy between Tadalafil and sildenafil, with greater patient and partner preference for Tadalafil [15]. This highlights the need for real-world comparative data between Avanafil and Tadalafil, which the present study aims to address. A meta-analysis encompassing 16 trials comparing Tadalafil and sildenafil for ED treatment found that both medications offer similar levels of efficacy and rates of adverse events [15]. However, Tadalafil stood out for its significantly greater impact on psychological well-being. Additionally, both patients and their partners showed a preference for Tadalafil over sildenafil. Despite these differences, no significant variation was observed in terms of treatment adherence and persistence between the two drugs, indicating that both are equally viable options for long-term ED management [13,15].

A 2015 network meta-analysis that indirectly evaluated the efficacy and safety of various phosphodiesterase inhibitors (PDEIs) for ED found distinct differences among the drugs. Sildenafil was identified as having the highest efficacy but was also associated with the highest overall rate of adverse events. On the other hand, Tadalafil showed intermediate efficacy but had the lowest rate of adverse events among the drugs analyzed. Additionally, vardenafil 10 mg and Avanafil 100 mg were found to have similar rates of adverse events compared to sildenafil 50 mg, but both demonstrated significantly lower overall efficacy [16].

This study has several strengths, including its prospective design, which allows for the systematic collection of data over a defined period, and the direct comparison between two widely used PDE-5 inhibitors, Avanafil and Tadalafil. The inclusion of a well-matched cohort of patients with similar baseline characteristics enhances the reliability of the findings. Additionally, the use of validated outcome measures, such as the IIEF-EF scores and SEP profiles, provides robust and clinically relevant data.

However, the study also has limitations. The sample size, while calculated to achieve adequate power, remains relatively small and may limit the generalizability of the findings to broader populations. The study's duration of 12 weeks, although sufficient to observe initial treatment effects, may not capture long-term outcomes and safety profiles. A primary limitation of this study is the non-randomized allocation of treatment groups, which may introduce selection bias. The choice of medication was based on clinical judgment, which could potentially influence the outcomes observed. Partner satisfaction was assessed using a non-validated numeric rating scale, limiting interpretability. Furthermore, the reliance on self-reported data for sexual performance and satisfaction introduces the potential for reporting bias. Objective measures of erectile rigidity, such as the Erection Hardness Score (EHS), were not assessed in this study. Therefore, conclusions are based primarily on validated patient‐reported outcomes (IIEF and SEP), and objective rigidity was not directly compared between treatment groups. The duration of diabetes mellitus was not captured systematically in our data collection. As the chronicity of diabetes may influence ED severity and responsiveness to therapy, this factor could not be analyzed and should be considered in future studies. Partner satisfaction was assessed using a non-validated instrument and should be considered exploratory. Lastly, the study did not include a placebo control group, which might have provided additional context for interpreting the efficacy results. Additionally, repeated outcome measurements were compared using multiple pairwise statistical tests rather than a repeated-measures model, which may increase the risk of type I error. These statistical limitations should be considered when interpreting the magnitude and precision of treatment effects. To confirm these findings and investigate the long-term safety and effectiveness of these treatments, future randomized studies are necessary. These studies should include larger sample sizes, longer follow-up periods, and a placebo control group.

## Conclusions

Overall, this study confirms the efficacy and safety of both Avanafil and Tadalafil for treating ED, with Tadalafil demonstrating statistically significant improvement in IIEF-EF scores. However, the choice between these medications should consider individual patient preferences, tolerability, and specific clinical situations, as both drugs offer substantial benefits with minimal risk of severe adverse effects. Future research with a larger sample population and longer follow-up periods may help to establish any subtle differences in efficacy and safety between these two treatments.
